# Mechanisms and Emerging Regulators of Neuroinflammation: Exploring New Therapeutic Strategies for Neurological Disorders

**DOI:** 10.3390/cimb47010008

**Published:** 2024-12-26

**Authors:** Mi Eun Kim, Jun Sik Lee

**Affiliations:** Immunology Research Lab & BK21-Four Educational Research Group for Age-Associated Disorder Control Technology, Department of Biological Science, Chosun University, Gwangju 61452, Republic of Korea; kimme0303@chosun.ac.kr

**Keywords:** neuroinflammation, microglia, astrocytes, therapeutic strategies

## Abstract

Neuroinflammation is a complex and dynamic response of the central nervous system (CNS) to injury, infection, and disease. While acute neuroinflammation plays a protective role by facilitating pathogen clearance and tissue repair, chronic and dysregulated inflammation contributes significantly to the progression of neurodegenerative disorders such as Alzheimer’s disease, Parkinson’s disease, and Multiple Sclerosis. This review explores the cellular and molecular mechanisms underlying neuroinflammation, focusing on the roles of microglia, astrocytes, and peripheral immune cells. Key signaling pathways, including NF-κB, JAK-STAT, and the NLRP3 inflammasome, are discussed alongside emerging regulators such as non-coding RNAs, epigenetic modifications, and the gut–brain axis. The therapeutic landscape is evolving, with traditional anti-inflammatory drugs like NSAIDs and corticosteroids offering limited efficacy in chronic conditions. Immunomodulators, gene and RNA-based therapeutics, and stem cell methods have all shown promise for more specific and effective interventions. Additionally, the modulation of metabolic states and gut microbiota has emerged as a novel strategy to regulate neuroinflammation. Despite significant progress, challenges remain in translating these findings into clinically viable therapies. Future studies should concentrate on integrated, interdisciplinary methods to reduce chronic neuroinflammation and slowing the progression of neurodegenerative disorders, providing opportunities for revolutionary advances in CNS therapies.

## 1. Introduction

Neuroinflammation is a complex and multifaceted response of the central nervous system (CNS) to injury, infection, or disease [1,2]. Acute neuroinflammation plays a critical role in preserving CNS homeostasis by facilitating pathogen clearance and promoting tissue repair. However, chronic and dysregulated neuroinflammation is progressively recognized as a central driver of neurodegenerative disorders, including Alzheimer’s disease, Parkinson’s disease, and Multiple Sclerosis [1,3,4]. These disorders are characterized by sustained activation of immune cells within the CNS, aberrant signaling pathways, and the infiltration of peripheral immune cells, leading to progressive neuronal damage and functional impairment [5,6].

Recent advances in the understanding of neuroinflammation have unveiled a dynamic interplay between various cellular components, including microglia, astrocytes, and peripheral immune cells. These cells mediate the delicate balance between neuroprotective and neurotoxic responses, influenced by molecular regulators such as transcription factors, cytokines, and signaling pathways [7,8,9,10]. However, the traditional view of neuroinflammation as a static process has shifted, with emerging evidence highlighting novel regulators such as non-coding RNAs, epigenetic modifications, and metabolic states that shape the inflammatory landscape of the CNS.

This evolving knowledge presents an opportunity to redefine therapeutic strategies for neuroinflammation, focusing on targeted interventions that address its molecular and cellular underpinnings. Exploring these mechanisms offers the potential to mitigate chronic inflammation while preserving its protective aspects, paving the way for innovative therapies to combat the progression of neurological disorders. This review aims to summarize current insights into the mechanisms and emerging regulators of neuroinflammation and to evaluate new therapeutic strategies that hold promise for transforming the treatment landscape for neurological diseases.

## 2. Mechanisms of Neuroinflammation

In this section, we will discuss the key cell types involved, the molecular pathways that mediate neuroinflammatory responses, and the complex interplay between the CNS and the peripheral immune system (Table 1).

### 2.1. Key Cellular Players in Neuroinflammation

Among the cellular components of neuroinflammation, microglia play a pivotal role as the resident immune cells in the CNS. In their resting state, microglia actively survey the microenvironment for signs of injury or infection [11,12]. Upon activation by stimuli such as amyloid beta plaques in Alzheimer’s disease or alpha synuclein aggregates in Parkinson’s disease, microglia can adopt a spectrum of phenotypes [7]. These range from pro-inflammatory states, characterized by the release of cytokines such as TNF-α, IL-1β, and IL-6, to anti-inflammatory states, which are marked by the production of IL-10 and TGF-β. This functional plasticity underscores their critical role in either exacerbating neuronal damage or promoting tissue repair [13,14,15]. Molecular regulators such as NF-κB drive pro-inflammatory responses, while pathways involving PPAR-γ and STAT6 support anti-inflammatory activities [16]. Dysregulation in this balance, particularly sustained activation of the NF-κB pathway, has been implicated in chronic neuroinflammation and progressive neuronal loss in neurodegenerative diseases [17].

#### 2.1.1. Microglia

Microglia are the resident immune cells in the CNS and are central to the neuroinflammatory process. In their resting state, microglia continuously monitor the CNS environment for signs of infection or injury [11]. Upon activation by various stimuli such as damaged neurons, amyloid beta plaques in Alzheimer’s disease, or alpha synuclein aggregates in Parkinson’s disease, microglia rapidly shift into an activated state, adopting different phenotypes that can be either neuroprotective or neurotoxic [7,18].

Activated microglia (M1 phenotype) produce pro-inflammatory cytokines such as TNF-α, IL-1β, and IL-6, which promote inflammation and contribute to neuronal injury. They also release reactive oxygen species (ROS) and nitric oxide (NO), further exacerbating oxidative stress and neuronal damage [3,19]. However, microglia can also adopt an anti-inflammatory or “M2” phenotype that help resolve inflammation and support tissue repair by secreting anti-inflammatory cytokines like IL-10 and TGF-β. The balance between these opposing states is critical; prolonged or uncontrolled pro-inflammatory microglial activation is a hallmark of neurodegenerative diseases [20]. Recent studies have identified several molecular switches that regulate microglial activation. The transcription factor NF-κB is a key driver of pro-inflammatory responses, while anti-inflammatory pathways, such as those mediated by STAT6, promote tissue repair. Targeting these regulatory pathways is an emerging area of research aimed at shifting microglia from a neurotoxic to a neuroprotective state [21,22,23].

#### 2.1.2. Astrocytes

Astrocytes, the most abundant glial cells in the CNS, also play a significant role in neuroinflammation. Traditionally considered as support cells for neurons, astrocytes are now recognized as active participants in the immune response. Like microglia, astrocytes can become reactive in response to injury or disease, adopting either pro-inflammatory or anti-inflammatory phenotypes [24,25].

Activated astrocytes release a range of cytokines, chemokines, and growth factors that influence the function of other CNS cells, including neurons and microglia. The pro-inflammatory activation of astrocytes is often characterized by the production of cytokines such as IL-1, IL-6, and TNF-α, as well as chemokines that attract peripheral immune cells to the CNS. These reactive astrocytes can contribute to the formation of glial scars, which, while initially protective, can hinder tissue regeneration and exacerbate neurodegeneration in the long term [7,24].

Astrocytes and microglia have an intimate relationship, and this interaction is essential for controlling neuroinflammatory responses. A cycle of feedback that increases inflammation is produced when microglia are activated, releasing substances that cause astrocytes to act on their pro-inflammatory properties. It has been demonstrated that IL-1α, TNF-α, and complement component 1q (C1q) are necessary and sufficient to produce reactive astrocyte phenotypes, therefore making them particularly important among these factors. Often called “A1 astrocytes”, these reactive astrocytes have neurotoxic qualities that lead to neuronal death and dysfunction. In order to ready astrocytes for a reactive transformation, IL-1α modifies their transcriptional responses, making them more sensitive to other inflammatory signals. In astrocytes, TNF-α and IL-1α interact in collaboration to upregulate pro-inflammatory pathways and increase the production of cytokines, chemokines, and reactive oxygen species. Furthermore, an important component of the complement cascade, C1q, interacts directly with astrocytes to stimulate the expression of genes linked to the A1 phenotype. When taken together, these microglial-derived substances induce astrocytes to develop a transcriptome and functional change, changing from homeostatic, supporting cells to neurotoxic ones that promote neuroinflammation and neuronal damage [26].

#### 2.1.3. Peripheral Immune Cells

Although the CNS is traditionally viewed as immune-privileged, it can be infiltrated by peripheral immune cells under pathological conditions. Disruption of the blood–brain barrier (BBB) allows immune cells such as T cells, B cells, and macrophages to enter the CNS and participate in the neuroinflammatory response [4,27]. In autoimmune disorders like Multiple Sclerosis, autoreactive T cells cross the BBB, triggering widespread inflammation and demyelination. Peripheral macrophages, when recruited to the CNS, can further contribute to neuroinflammation by releasing pro-inflammatory cytokines and phagocytosing neuronal debris [28,29]. Recent studies have also highlighted the role of T regulatory cells (Tregs) in controlling neuroinflammation. Tregs can limit the immune response by suppressing the activation of effector T cells and producing anti-inflammatory cytokines like IL-10. Therapeutic strategies aimed at enhancing Treg functions are currently under investigation for their potential to reduce neuroinflammation in conditions such as Multiple Sclerosis [30]. Moreover, Autoreactive T cells play a particularly destructive role in conditions such as Multiple Sclerosis, where Th1 and Th17 subsets drive demyelination through the secretion of IFN-γ and IL-17. Conversely, Tregs mitigate inflammation by suppressing effector T cell activity and producing anti-inflammatory cytokines like IL-10 and TGF-β. Enhancing Treg functions has emerged as a promising therapeutic strategy to reduce neuroinflammation in autoimmune and neurodegenerative diseases [31].

**Table 1:** Key Cellular components and their roles in neuroinflammation.

Component	Role	Key Features	Ref
Microglia	Primary immune cells in CNS	Respond to amyloid beta, alpha synuclein, and injury; pro-inflammatory (M1) and anti-inflammatory (M2) phenotypes	[11,20]
Astrocytes	Support neurons and modulate neuroinflammation	Pro-inflammatory (A1) vs. anti-inflammatory (A2); secrete cytokines (IL-1, IL-6) and chemokines	[24,25]
Peripheral Cells	Mediate immune–CNS interactions during BBB disruption	T cells and macrophages contribute to inflammation; Tregs regulate responses via IL-10 and TGF-β	[27,28,29,30]

### 2.2. Molecular Pathways Driving Neuroinflammation

**Figure 1:**
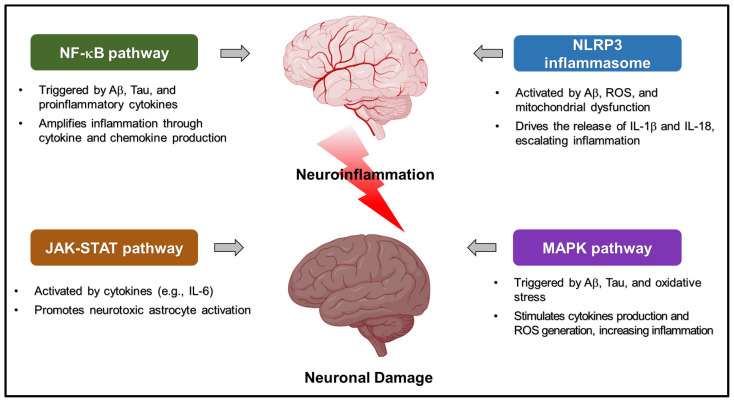
The major molecular pathways driving neuroinflammation in Alzheimer’s disease, highlighting their interconnected roles in perpetuating chronic inflammation. At the center of the diagram is neuroinflammation, which is fueled by four key pathways. The NF-κB pathway, activated by amyloid beta (Aβ) and tau proteins, promotes the production of pro-inflammatory cytokines such as TNF-α and IL-6, amplifying the inflammatory response. The NLRP3 inflammasome, triggered by Aβ, reactive oxygen species (ROS), and mitochondrial dysfunction, leads to the release of IL-1β and IL-18, further escalating neuroinflammation. The JAK-STAT pathway, induced by cytokines like IL-6, drives the activation of neurotoxic astrocytes, which contribute to neuronal damage. Finally, the MAPK pathway, stimulated by Aβ, tau, and oxidative stress, enhances ROS production and cytokine release, exacerbating oxidative damage and inflammation. Together, these pathways form a complex network that underpins the inflammatory processes observed in Alzheimer’s disease.

Neuroinflammation is orchestrated by a variety of molecular signaling pathways that regulate the activation and function of immune cells in the CNS. Key pathways include the NF-κB signaling cascade, the JAK-STAT pathway, the NLRP3 inflammasome, and MAPK signaling [32]. Each of these pathways can be activated by different stimuli, including infections, misfolded proteins, and cellular stress (Table 2, Figure 1).

#### 2.2.1. NF-κB Signaling Pathway

The nuclear factor kappa-light-chain-enhancer of activated B cells (NF-κB) pathway is one of the most prominent regulators of inflammation in both the peripheral and central immune systems. In the CNS, the activation of the NF-κB pathway in microglia, astrocytes, and even neurons leads to the production of pro-inflammatory cytokines, chemokines, and adhesion molecules that recruit immune cells and amplify the inflammatory response [24,33]. The activation of the NF-κB pathway can be triggered by a variety of signals, including Toll-like receptors (TLRs), TNF-α, and IL-1β. Dysregulation of this pathway contributes to chronic inflammation in neurodegenerative diseases. For example, excessive NF-κB activation has been implicated in Alzheimer’s disease, where it promotes the production of amyloid beta plaques and tau pathology. Targeting NF-κB signaling represents a promising strategy for reducing neuroinflammation and slowing disease progression [16].

#### 2.2.2. JAK-STAT Pathway

The Janus kinase–signal transducer and activator of transcription (JAK-STAT) pathway represents another key molecular mechanism in neuroinflammation [34]. Cytokines such as IL-6 and IL-10 activate the JAK-STAT pathway, resulting in transcriptional programs that influence immune cell activation, proliferation, and survival. In the CNS, STAT3 activation in astrocytes has been implicated in the induction of the neurotoxic A1 astrocyte phenotype, which exacerbates neuronal damage [35]. Therapeutically, inhibitors of the JAK-STAT pathway, such as Ruxolitinib, are currently under investigation for their ability to mitigate neuroinflammation in diseases such as Multiple Sclerosis and amyotrophic lateral sclerosis [36].

#### 2.2.3. NLRP3 Inflammasome

The NLRP3 inflammasome is a multiprotein complex that plays a central role in the innate immune response. Upon activation by signals such as ATP, ROS, or misfolded proteins, the NLRP3 inflammasome triggers the activation of caspase-1. This process leads to the maturation and release of pro-inflammatory cytokines IL-1β and IL-18. The NLRP3 inflammasome is highly active in microglia during neuroinflammation. It is implicated in a range of neurodegenerative diseases, including Alzheimer’s and Parkinson’s [37]. In Alzheimer’s disease, for instance, amyloid beta plaques activate the NLRP3 inflammasome, contributing to chronic inflammation and neuronal damage. The pharmacological inhibition of the NLRP3 inflammasome is being actively investigated as a therapeutic strategy to mitigate neuroinflammation in these diseases [37,38,39].

#### 2.2.4. MAPK Signaling Pathway

The mitogen-activated protein kinase (MAPK) signaling pathway is a highly conserved mechanism that regulates cellular responses to various stressors, including inflammation, oxidative stress, and infection. In the central nervous system (CNS), the MAPK pathway plays a critical role in modulating neuroinflammation by influencing the production of pro-inflammatory cytokines, chemokines, and other immune mediators. Its activation is particularly relevant during microglial and astrocytic responses, which contribute significantly to the progression of neurodegenerative diseases such as Alzheimer’s disease, Parkinson’s disease, and Multiple Sclerosis. The MAPK pathway is organized into three main branches—ERK (extracellular signal-regulated kinase), JNK (c-Jun N-terminal kinase), and p38 MAPK—each of which is activated by distinct stimuli and mediates specific cellular outcomes. Among these, the p38 MAPK pathway has been extensively studied in relation to neuroinflammation, as it is directly activated by inflammatory cytokines such as IL-1β and TNF-α, as well as by oxidative stress and misfolded proteins. Upon activation, the p38 MAPK pathway triggers the expression of pro-inflammatory cytokines, including IL-6 and TNF-α, and enhances the activity of inflammasomes like NLRP3, further amplifying the inflammatory response. Microglial activation during neuroinflammation is heavily influenced by the MAPK pathway. In particular, p38 MAPK activation in microglia promotes the production of reactive oxygen species (ROS) and pro-inflammatory cytokines, which exacerbate neuronal damage. Similarly, the JNK pathway is activated in microglia under stress conditions, driving apoptosis and releasing neurotoxic factors. In astrocytes, MAPK signaling plays an equally critical role. The activation of the p38 MAPK and JNK pathways in astrocytes leads to the production of cytokines, chemokines, and growth factors that perpetuate a cycle of inflammation by further stimulating microglia and recruiting peripheral immune cells. While the ERK pathway in astrocytes is less understood, it is thought to contribute to astrocytic proliferation and scar formation in response to CNS injury. In the context of neurodegenerative diseases, aberrant MAPK signaling is a key driver of chronic inflammation and neuronal dysfunction. In Alzheimer’s disease, amyloid beta and tau proteins activate the p38 MAPK pathway in both microglia and astrocytes, resulting in the sustained production of pro-inflammatory cytokines such as IL-1β and TNF-α. This chronic activation contributes to synaptic dysfunction, oxidative stress, and eventual neuronal loss. Similarly, in Parkinson’s disease, alpha synuclein aggregates activate the p38 MAPK and JNK pathways, triggering microglial activation and dopaminergic neuron loss. In Multiple Sclerosis, MAPK signaling facilitates the secretion of chemokines that attract peripheral immune cells into the CNS, exacerbating demyelination and axonal damage. Given its central role in neuroinflammation, the MAPK pathway has emerged as a promising therapeutic target. Preclinical studies have demonstrated that the inhibition of MAPK signaling can reduce inflammation and neuronal damage in models of Alzheimer’s disease, Parkinson’s disease, and Multiple Sclerosis. Small-molecule inhibitors targeting p38 MAPK, such as SB203580, have shown efficacy in suppressing cytokine production and reducing oxidative stress. Similarly, JNK inhibitors like SP600125 have been explored for their ability to prevent microglial-induced neurotoxicity and apoptosis. Furthermore, novel dual-target inhibitors that simultaneously block p38 MAPK and JNK pathways are being developed to achieve more comprehensive suppression of inflammation. Several MAPK inhibitors are currently in clinical trials, with the aim of translating these findings into effective treatments for neurodegenerative diseases [40,41].

**Table 2:** Major molecular pathways involved in neuroinflammatory responses.

Pathway	Trigger	Key Action	Implications in Disease	Ref
NF-κB	Toll-like receptors (TLRs), TNF-α	Induces cytokines and chemokine production, and amplifies inflammation	Contributes to amyloid-beta and tau pathology in Alzheimer’s disease	[16,24]
JAK-STAT	Cytokines (IL-6, IL-10)	Regulates immune cell proliferation, and promotes neurotoxic phenotypes	Linked to A1 astrocyte activation in neurodegeneration	[34,35]
NLRP3 Inflammasome	ROS, ATP, misfolded proteins	Activates caspase-1, and releases IL-1β and IL-18	Exacerbates inflammation in Alzheimer’s and Parkinson’s diseases	[37,38,39]
MAPK	Inflammatory stimuli	Regulates stress responses and cytokine production	Activated by amyloid beta, promotes pro-inflammatory states in microglia	[40,41]

## 3. Emerging Regulators of Neuroinflammation

The complexity of neuroinflammation extends beyond classic immune and cellular mechanisms, with emerging evidence highlighting the role of non-coding RNAs, epigenetic modifications, the gut–brain axis, and metabolic states as critical regulators. These novel insights not only deepen our understanding of neuroinflammation but also present promising therapeutic targets for neurodegenerative diseases (Table 3).

### 3.1. MicroRNAs and IncRNAs

MicroRNAs (miRNAs) and long non-coding RNAs (lncRNAs) have emerged as pivotal post-transcriptional regulators of neuroinflammation. These non-coding RNAs modulate gene expression by interacting with messenger RNAs (mRNAs) or transcriptional machinery, influencing the activity of immune cells within the CNS.

MicroRNAs: Specific miRNAs such as miR-155 and miR-146a have been extensively studied for their roles in neuroinflammation. miR-155 is upregulated in activated microglia and astrocytes, promoting the production of pro-inflammatory cytokines like TNF-α and IL-1β. miR-155, encoded by *MIR155HG* on chromosomes, is a pro-inflammatory miRNA upregulated during immune activation, promoting inflammation by targeting negative regulators like SOCS1, SHIP1, and IL13Rα1. These targets enhance cytokine production and sustain microglial and astrocytic activation. In contrast, miR-146a, encoded by *MIR146A* on chromosomes, acts as an anti-inflammatory regulator by suppressing NF-κB signaling through targets like TRAF6, IRAK1, and CARD10, thereby mitigating excessive inflammation and restoring immune balance. Dysregulation of these miRNAs is linked to neurodegenerative diseases, highlighting their potential as therapeutic targets. Therapeutically, strategies to inhibit miR-155 or enhance miR-146a expression are being explored to restore immune homeostasis. Dysregulated miRNA expression has been linked to neurodegenerative conditions, with miR-155 overexpression observed in Alzheimer’s disease and Multiple Sclerosis [42].

Long Non-Coding RNAs: LncRNAs such as NEAT1 and MALAT1 are implicated in the regulation of neuroinflammatory pathways. NEAT1 promotes inflammasome activation in microglia by enhancing NLRP3 transcription, exacerbating neuroinflammation in conditions like Parkinson’s disease. Targeting these non-coding RNAs has been proposed as a therapeutic strategy to restore balance in inflammatory signaling pathways [43].

### 3.2. Epigenetic Modifications

Epigenetic modifications, including DNA methylation, histone modifications, and chromatin remodeling, play crucial roles in regulating gene expression during neuroinflammation. These modifications provide a dynamic mechanism for integrating environmental and pathological signals, shaping the inflammatory response.

DNA Methylation: Altered DNA methylation patterns in the promoters of inflammatory genes have been observed in neuroinflammatory conditions. For instance, the hypomethylation of the IL-6 promoter in microglia correlates with increased cytokine expression during neurodegeneration [44].

**Table 3:** Novel regulators shaping neuroinflammatory mechanisms.

Regulator	Mechanism	Implications	Ref
MicroRNAs (e.g., miR-155, miR-146a)	Post-transcriptional regulation of inflammatory gene expression	Dysregulation linked to Alzheimer’s disease and Multiple Sclerosis.	[42]
lncRNAs (e.g., NEAT1)	Modulates inflammasome activation and enhances inflammatory signaling	Associated with Parkinson’s disease pathology.	[43]
Epigenetic Modifications	DNA methylation, histone modifications, chromatin remodeling	Influence inflammatory gene expression and microglial activity.	[45,46,47,48]
Gut–Brain Axis	Microbial metabolites (e.g., SCFAs) and alpha synuclein aggregation	Dysbiosis linked to Parkinson’s disease and neuroinflammatory diseases.	[49,50]
Metabolic States	Shift between glycolysis and oxidative phosphorylation in microglia	Regulates pro- and anti-inflammatory phenotypes.	[51,52,53]

Histone Modifications: Post-translational modifications of histones, such as acetylation and methylation, modulate the accessibility of inflammatory gene loci. Histone acetyltransferases (HATs) enhance the transcription of pro-inflammatory genes, while histone deacetylases (HDACs) suppress their expression. Pharmacological inhibitors of HDACs, such as Vorinostat, have shown promise in reducing inflammation in preclinical models of Multiple Sclerosis [46,47]. However, there has only been a limited amount of success in making these therapeutic applications credible. The physiological roles of HDACs in particular are currently poorly understood. Therefore, both the advantages of harnessing HDACs and the risks of targeting them should be further explored.

Chromatin Remodeling: Chromatin remodeling, particularly through the polycomb repressive complex 2 (PRC2) and its catalytic subunit EZH2, plays a vital role in maintaining microglial homeostasis and regulating neuroinflammation. PRC2 suppresses inflammatory gene expression by tri-methylating histone H3 at lysine 27 (H3K27me3), leading to chromatin compaction and transcriptional repression. A loss of PRC2 function or EZH2 dysregulation are associated with heightened microglial activation, increased pro-inflammatory cytokine production, and exacerbated neurodegeneration. Targeting PRC2 pathways with EZH2 inhibitors, such as DZNep or GSK-126, has shown promise in reducing neuroinflammation and mitigating microglial activation, highlighting the therapeutic potential of chromatin remodeling in neurodegenerative diseases [48].

### 3.3. Gut–Brain Axis

The gut–brain axis has garnered significant attention as a bidirectional communication network that influences CNS homeostasis and neuroinflammatory responses. The composition and activity of gut microbiota are critical regulators of this axis.

Microbiota and Immune Modulation: Gut dysbiosis is associated with an altered production of microbial metabolites such as short-chain fatty acids (SCFAs), which regulate microglial activation and peripheral immune cell infiltration into the CNS. By preventing NF-κB activation in microglia and lowering the synthesis of pro-inflammatory cytokines, butyrate is one of these metabolites that has a significant anti-inflammatory effect. Additionally, butyrate supports the development and functionality of regulatory T cells (Tregs), which inhibit peripheral immunological activation and CNS infiltration. SCFAs are important participants in the gut–brain axis because of their dual function, which involves regulating both peripheral and central nervous system immune responses [49]. Alpha synuclein aggregates from the gut are thought to be transported to the brain in Parkinson’s disease through the vagus nerve, which is an immediate pathway for communication between the gut and central nervous system. These clusters set off a chain reaction of neuroinflammation in the brain by activating microglia. Research has indicated that individuals with Parkinson’s disease frequently have a greater amount of pro-inflammatory bacteria such Enterobacteriaceae and lower levels of bacteria that produce SCFAs. Systemic immunological activation and increased gut inflammation are related to this microbial imbalance. Preclinical models have demonstrated promise in restoring microbial balance with therapies such fecal microbiota transplantation (FMT), probiotics, and prebiotics. For instance, it has been demonstrated that probiotics such as Bifidobacterium and Lactobacillus species reduce inflammation by improving the generation of SCFAs and modifying immune responses. In animal models of Parkinson’s disease, FMT from healthy donors has also shown promise in lowering neuroinflammation and easing motor symptoms [50,54].

### 3.4. Metabolism and Inflammation

Metabolic states profoundly influence neuroinflammatory processes, with immune cells within the CNS responding dynamically to changes in glucose metabolism, oxidative stress, and mitochondrial function.

Glucose Metabolism: The metabolic reprogramming of microglia toward glycolysis during activation supports the rapid production of pro-inflammatory cytokines. Normally, at rest, microglia generate energy mostly by oxidative phosphorylation (OXPHOS), an efficient process for producing ATP. But when microglia are activated, their metabolic profile changes to aerobic glycolysis, which is referred to as the Warburg effect. Pro-inflammatory processes, such as the production of cytokines, require a lot of energy, which is supported by this metabolic turn. The metabolic states of microglia are dynamically modified according to their activation status. In the pro-inflammatory (M1) state, microglia use glycolysis to generate energy and facilitate the production of cytokines such as TNF-α, IL-1β, and IL-6. Intermediates like succinate stabilize HIF-1α and increase NLRP3 inflammasome activity, which intensifies inflammation. On the other hand, oxidative phosphorylation (OXPHOS), which effectively produces ATP, is primarily used by microglia and astrocytes in the anti-inflammatory (M2) state. This is similar to processes like tissue repair and the release of anti-inflammatory cytokines like IL-10 and TGF-β [51,52].

Oxidative Stress: Inflammasomes and NF-κB signaling pathways are activated by reactive oxygen species (ROS) produced during mitochondrial dysfunction, which contribute significantly to the acceleration of neuroinflammation. With the success of several FDA-approved drugs, oxidative stress management has become a crucial therapeutic approach for neurological disorders. One of the most effective free radical scavengers among them is edaravone (Radicava), which is authorized to treat ALS. Edaravone reduces oxidative stress by neutralizing ROS, which reduces inflammation and cellular damage that cause dementia. Scavenging hydroxyl radicals, stopping lipid peroxidation, and maintaining cellular integrity are all part of its mechanism. According to clinical research, edaravone, especially when given early in the course of the disease, slows the progression of physical decline in ALS patients [53].

## 4. Current Therapeutic Strategies for Neuroinflammation

The treatment of neuroinflammation presents a significant challenge due to its dual role in the CNS—both protective and deleterious. Current therapeutic strategies aim to mitigate chronic and dysregulated inflammation while preserving its beneficial aspects. This section examines the traditional and emerging approaches for addressing neuroinflammation, including anti-inflammatory drugs, immunomodulators, gene and RNA-based therapies, and stem cell therapies, alongside their potential limitations (Table 4).

### 4.1. Anti-Inflammatory Drugs

Nonsteroidal anti-inflammatory drugs (NSAIDs) and corticosteroids have been the cornerstone of anti-inflammatory therapy for decades. NSAIDs function by inhibiting cyclooxygenase (COX) enzymes, thereby reducing the synthesis of pro-inflammatory prostaglandins. Although NSAIDs have shown some promise in reducing neuroinflammation, particularly in early-stage Alzheimer’s disease, their efficacy in chronic neurodegenerative conditions has been inconsistent. Long-term use is also associated with gastrointestinal, renal, and cardiovascular side effects [55,56].

Corticosteroids, which suppress inflammation through the inhibition of NF-κB and other inflammatory pathways, are effective in acute settings, such as traumatic brain injury or acute exacerbations of Multiple Sclerosis. However, their chronic use is limited by significant adverse effects, including immunosuppression, osteoporosis, and metabolic disturbances. Furthermore, their inability to cross the blood–brain barrier (BBB) effectively limits their therapeutic potential for CNS-specific inflammation [57,58].

### 4.2. Immunomodulators

Emerging immunomodulatory drugs are designed to target specific pathways within the neuroinflammatory cascade, offering more precise therapeutic effects.

Anti-TNF Therapies: Tumor necrosis factor alpha (TNF-α) is a central cytokine in neuroinflammation, driving microglial activation and neuronal damage. Anti-TNF drugs such as infliximab and etanercept have shown efficacy in peripheral inflammatory diseases like rheumatoid arthritis, but their efficacy in the CNS has been limited due to poor BBB penetration. Innovative approaches, such as intrathecal administration, are under investigation to enhance their CNS delivery [59,60].

IL-6 Inhibitors: IL-6 plays a significant role in CNS inflammation and neurodegeneration. Tocilizumab, an IL-6 receptor antagonist, has shown promise in preclinical models of neuroinflammation. Recent studies suggest that targeting IL-6 signaling may attenuate inflammation in conditions such as Multiple Sclerosis and Alzheimer’s disease, though clinical trials are ongoing to validate these findings [61,62].

Small Molecule Inhibitors: Janus kinase (JAK) inhibitors, such as ruxolitinib, target the JAK-STAT pathway, which regulates the expression of inflammatory genes. These inhibitors are currently being tested in neuroinflammatory conditions and have demonstrated anti-inflammatory effects in preclinical models of Multiple Sclerosis and ALS [63,64].

### 4.3. Gene and RNA-Based Therapies

Gene and RNA-based therapies are rapidly emerging as powerful tools for modulating neuroinflammation with high specificity.

RNA Interference (RNAi): RNAi technology utilizes small interfering RNAs (siRNAs) to silence the expression of pro-inflammatory genes. In order to reduce the production of inflammatory proteins, siRNAs are delivered into cells, where they bind to and eliminate complementary mRNA. By reducing cytokine production as well as decreasing microglial activation, siRNAs that target TNF-α or NLRP3 inflammasome components, for example, have shown significant effectiveness in preclinical models of Parkinson’s and Alzheimer’s diseases. The stability and bioavailability of siRNAs have also been enhanced by developments in delivery vehicles, including viral vectors and lipid nanoparticles, which allow for the efficient targeting of CNS cells. The effects of off-target and immunological activation are still important issues that require more study and further improvements despite these developments [65,66].

CRISPR-Cas9: The CRISPR-Cas9 system allows for precise gene editing to either suppress pro-inflammatory mediators or enhance anti-inflammatory pathways. Preclinical studies have demonstrated the potential of CRISPR-Cas9 in modulating microglial activation and reducing inflammatory cytokine production. Pro-inflammatory mediators might be suppressed or anti-inflammatory pathways may become stronger according to this technology. The Cas9 nuclease can disrupt pathogenic genes or allow the insertion of therapeutic sequences by introducing double-stranded breaks at specified DNA sequences using a guide RNA (gRNA). Using preclinical research, CRISPR-Cas9 can decrease the synthesis of inflammatory cytokines including IL-6 and IL-1β and modify microglial activation. The technique was also applied to target components of the NLRP3 inflammasome, which has been shown to significantly decrease neuroinflammation in mice with Alzheimer’s disease. Despite the remarkable precision of CRISPR-Cas9, there are challenges to its practical application, such as the possibility of off-target effects and transport to CNS cells [67,68].

Antisense Oligonucleotides (ASOs): ASOs bind to specific mRNA sequences, blocking their translation. This approach has been used to target misfolded proteins in neurodegenerative diseases, such as tau in Alzheimer’s disease, while also reducing inflammatory mediators. Nusinersen (Spinraza), an FDA-approved ASO for spinal muscular atrophy, exemplifies the therapeutic potential of this technology in CNS disorders. Nusinersen enhances motor function and survival in SMA patients by targeting the SMN2 gene, demonstrating the therapeutic potential of ASOs in CNS diseases. ASOs are being investigated to inhibit cytokines such as TNF-α and IL-6 in neurological disorders as a result of this technology’s success. ASOs are a promising treatment for chronic neuroinflammation because of their high specificity and relatively low immunogenicity; nevertheless, problems of cost and CNS administration still need to be resolved [69,70].

### 4.4. Stem Cell Therapies

Stem cell-based therapies represent a promising frontier for addressing neuroinflammation and promoting CNS repair. Mesenchymal stem cells (MSCs) and induced pluripotent stem cells (iPSCs) are particularly noteworthy for their immunomodulatory properties and potential to differentiate into neural cells.

Immunomodulation: MSCs secrete anti-inflammatory cytokines such as IL-10 and TGF-β, which suppress microglial and astrocytic activation. They also promote the expansion of regulatory T cells (Tregs), reducing peripheral immune infiltration into the CNS. In preclinical models of Multiple Sclerosis, MSC transplantation has demonstrated a significant reduction in neuroinflammation and demyelination [71,72,73].

Tissue Repair: iPSCs offer the potential to replace damaged neurons and glial cells. Despite their immunomodulatory effects being less well-characterized than those of MSCs, iPSCs have shown promise in regenerating neural tissue in models of Parkinson’s disease and spinal cord injury. However, challenges remain in ensuring their safety and preventing tumorigenesis [74,75,76].

**Table 4:** Existing and emerging therapeutic approaches for neuroinflammation.

Strategy	Example Drugs	Mechanism	Limitations	Ref
Anti-inflammatory Drugs	NSAIDs (e.g., Ibuprofen, Aspirin)	Inhibit COX enzymes and reduce prostaglandin synthesis	GI toxicity, poor BBB penetration, limited efficacy in chronic conditions.	[53,55]
Corticosteroids	Dexamethasone, Methylprednisolone	Suppress NF-κB signaling and promote anti-inflammatory mediators	Systemic side effects, limited CNS-specificity, rebound inflammation risk.	[56]
Immunomodulators	Anti-TNF (Infliximab), IL-6 inhibitors	Block inflammatory cytokines and pathways	Poor BBB penetration, limited long-term data for CNS applications.	[58,59,60,61,62,63,64]
Gene and RNA Therapies	siRNA, CRISPR, ASOs	Target specific genes involved in inflammation and misfolded proteins	High specificity; challenges with delivery, off-target effects.	[65,66,67,68,69,70]
Stem Cell Therapies	MSCs, iPSCs	Modulate immune responses, promote repair	Tumorigenesis risk, safety concerns, and regulatory hurdles.	[71,72,73,74,75,76]

## 5. Discussion

The findings presented in this review highlight the multifaceted nature of neuroinflammation and its critical role in the progression of neurological disorders. Chronic neuroinflammation emerges as a key driver of neurodegeneration, fueled by the sustained activation of microglia, astrocytes, and peripheral immune cells [1,77,78]. These cellular responses are orchestrated by complex molecular pathways, including NF-κB, JAK-STAT, the NLRP3 inflammasome, and MAPK signaling, which collectively amplify inflammatory responses and exacerbate neuronal damage [3,32,79]. Importantly, emerging regulators such as non-coding RNAs, epigenetic modifications, metabolic states, and the gut–brain axis add further layers of complexity, offering novel insights into the dynamic regulation of CNS inflammation [80,81].

Despite the progress in our understanding of the mechanisms of neuroinflammation, therapeutic interventions remain a significant challenge. Traditional anti-inflammatory drugs, including NSAIDs and corticosteroids, have demonstrated limited efficacy in chronic neurodegenerative conditions due to their inability to target CNS-specific inflammation and their systemic side effects [82,83,84]. While these drugs are effective in managing acute inflammation, their role in long-term disease modification is minimal. This underscores the need for more targeted approaches that can penetrate the blood–brain barrier (BBB) and selectively modulate inflammatory pathways [85,86].

Developing immunomodulatory strategies represents a promising advancement in the field. Drugs targeting TNF-α and IL-6, as well as JAK-STAT inhibitors, have shown potential in preclinical models, though their clinical utility in CNS disorders remains to be fully validated [34]. Gene and RNA-based therapies, including RNA interference (RNAi), CRISPR-Cas9, and antisense oligonucleotides (ASOs), offer unparalleled precision in targeting specific inflammatory mediators and disease-driving genes [87]. However, challenges related to delivery, safety, and off-target effects must be addressed to translate these therapies into viable clinical treatments.

Stem cell therapies hold considerable promise for their dual ability to modulate inflammation and promote tissue repair. Mesenchymal stem cells (MSCs) and induced pluripotent stem cells (iPSCs) have demonstrated significant immunomodulatory and neuroregenerative effects in preclinical studies [88,89,90]. Nevertheless, concerns regarding their long-term safety, potential for tumorigenesis, and scalability remain barriers to widespread clinical adoption.

The evolving role of the gut–brain axis and metabolic reprogramming in neuroinflammation offers intriguing opportunities for novel therapeutic interventions [91]. Manipulating gut microbiota through probiotics, prebiotics, or fecal microbiota transplantation could potentially modulate systemic and CNS-specific immune responses. Similarly, targeting metabolic pathways that regulate microglial and astrocytic phenotypes may provide new avenues for reducing neuroinflammation and promoting neuronal survival [92,93,94].

Future research should prioritize the integration of multi-modal approaches that combine traditional therapies with emerging technologies. For instance, combining anti-inflammatory drugs with RNA-based therapies or stem cell treatments could achieve synergistic effects, enhancing both efficacy and precision. Additionally, leveraging advances in systems biology and bioinformatics will be crucial for identifying novel therapeutic targets and personalizing treatment strategies based on individual patient profiles.

## 6. Conclusions

In conclusion, while significant strides have been made in understanding and targeting neuroinflammation, its complexity necessitates innovative, multidisciplinary approaches. By addressing the underlying mechanisms and emerging regulators, it may be possible to not only mitigate inflammation but also slow or even reverse the progression of neurological disorders. This comprehensive approach holds the potential to transform the therapeutic landscape, offering hope for patients with currently intractable neurodegenerative diseases.

## Data Availability

The data presented in this study are available on request from the corresponding author. The data are not publicly available due to privacy.
